# Development of a Thymoquinone Polymeric Anticancer Nanomedicine through Optimization of Polymer Molecular Weight and Nanoparticle Architecture

**DOI:** 10.3390/pharmaceutics12090811

**Published:** 2020-08-27

**Authors:** Suhair Sunoqrot, Malek Alfaraj, Ala’a M. Hammad, Violet Kasabri, Dana Shalabi, Ahmad A. Deeb, Lina Hasan Ibrahim, Khaldoun Shnewer, Ismail Yousef

**Affiliations:** 1 Department of Pharmacy, Faculty of Pharmacy, Al-Zaytoonah University of Jordan, Amman 11733, Jordan; malek.fraaj@gmail.com (M.A.); alaa.hammad@zuj.edu.jo (A.M.H.); a.deeb@zuj.edu.jo (A.A.D.); linaibrahim9345@yahoo.com (L.H.I.); 2 Department of Biopharmaceutics and Clinical Pharmacy, School of Pharmacy, University of Jordan, Amman 11942, Jordan; v.kasabri@ju.edu.jo (V.K.); danashalabi.ju@gmail.com (D.S.); 3 Smart Medical Labs, Amman 11180, Jordan; khaldoun@clemjo.com (K.S.); info@smartlabs-jo.com (I.Y.)

**Keywords:** thymoquinone, polymeric nanoparticles, mPEG-PCL, anticancer nanomedicine, drug delivery

## Abstract

Thymoquinone (TQ) is a water-insoluble natural compound isolated from *Nigella sativa* that has demonstrated promising chemotherapeutic activity. The purpose of this study was to develop a polymeric nanoscale formulation for TQ to circumvent its delivery challenges. TQ-encapsulated nanoparticles (NPs) were fabricated using methoxy poly(ethylene glycol)-*b*-poly(ε-caprolactone) (mPEG-PCL) copolymers by the nanoprecipitation technique. Formulation variables included PCL chain length and NP architecture (matrix-type nanospheres or reservoir-type nanocapsules). The formulations were characterized in terms of their particle size, polydispersity index (PDI), drug loading efficiency, and drug release. An optimized TQ NP formulation in the form of oil-filled nanocapsules (F2-NC) was obtained with a mean hydrodynamic diameter of 117 nm, PDI of 0.16, about 60% loading efficiency, and sustained in vitro drug release. The formulation was then tested in cultured human cancer cell lines to verify its antiproliferative efficacy as a potential anticancer nanomedicine. A pilot pharmacokinetic study was also carried out in healthy mice to evaluate the oral bioavailability of the optimized formulation, which revealed a significant increase in the maximum plasma concentration (C_max_) and a 1.3-fold increase in bioavailability compared to free TQ. Our findings demonstrate that the versatility of polymeric NPs can be effectively applied to design a nanoscale delivery platform for TQ that can overcome its biopharmaceutical limitations.

## 1. Introduction

Cancer is considered one of the leading causes of death worldwide and is responsible for millions of deaths annually [1]. Therapeutic success with conventional chemotherapeutic agents is complicated by their notoriously severe side effects, rapid clearance, and tumor relapse due to onset of multidrug resistance [2]. This has created a need to find alternative therapeutics and drug delivery systems that can eliminate cancer patients’ tumor burden more effectively.

Throughout history, medicinal plants have been used to treat a wide range of diseases. Due to their structural diversity, natural products and their derivatives constitute a rich source of bioactive compounds with unique pharmacologic effects against various ailments including cancer [3]. *Nigella sativa* (black seed or black cumin) is an annual flowering plant endogenous to the Mediterranean region and parts of India and Pakistan [4]. Thymoquinone (2-isopropyl-5-methylbenzo-1,4-quinone; TQ) is the major constituent of the volatile oil of *N. sativa*. Since it was first isolated, TQ has been investigated for its therapeutic benefits such as antioxidant, anti-inflammatory, anti-diabetic, immunomodulatory, and anticancer effects, in both in vitro and in vivo settings [5]. Despite its prominent bioactivity, TQ has been faced with major biopharmaceutical challenges related to its poor aqueous solubility, hindering its therapeutic potential in vivo [6].

Nanotechnology has provided pharmaceutical scientists with versatile solutions that hold enormous promise to overcome the delivery challenges of hydrophobic bioactive natural products such as TQ [7]. Various types of nanocarriers have been explored in order to improve the therapeutic index, solubility, stability, circulation time, targeting efficacy, and bioavailability of poorly water-soluble drug candidates [8,9]. Several TQ-based nanocarrier systems have been reported in the literature. Many of these nanoscale formulations have been based on lipids, such as liposomes [10,11], solid lipid nanoparticles (NPs) [12], and nanostructured lipid carriers [13,14,15]. Inorganic NPs such as those based on gold [16] and silica [17] have also been investigated to improve the anticancer activity of TQ.

Polymeric nanocarriers represent a unique class of materials by virtue of their ability to be tailored for various biomedical applications depending on the choice of polymer and method of nanocarrier fabrication and drug incorporation. Fakhoury et al. reported the encapsulation of TQ into poly(styrene-*b*-ethylene oxide) NPs by flash nanoprecipitation [18]. The NP formulation exhibited equal cytotoxicity to MCF-7 cells and enhanced anticancer activity toward MDA-MB-231 cells compared to free TQ. Bhattacharya et al. utilized poly(ethylene glycol) (PEG) and poly(vinylpyrrolidone) to prepare TQ NPs by the solvent evaporation technique [19]. The NPs enhanced the anticancer activity of free TQ and mediated its anti-migratory effect on MCF-7 cells in vitro. TQ NPs were also able to protect tumor-bearing mice from cancer-induced systemic toxicity and hepatic damage. Another study by Soni et al. described the co-encapsulation of TQ and paclitaxel in poly(lactide-co-glycolide) (PLGA) NPs to achieve synergistic anticancer activity against MCF-7 cells [20].

In the present study, we aimed to design a polymeric NP formulation for TQ based on the copolymer methoxy poly(ethylene glycol)-*b*-(ε-caprolactone) (mPEG-PCL), and evaluate the NP formulation as a potential anticancer nanomedicine. Both PEG and PCL are FDA approved. In particular, PCL (alone or in the form of a PEGylated copolymer) has been widely used for the encapsulation of hydrophobic drugs due to its stability, controlled release properties, biodegradability, and biocompatibility [21,22,23]. mPEG-PCL can be synthesized with varying chain lengths of the biodegradable block (i.e., PCL) in order to modulate its physicochemical properties. It can also be used to prepare NPs with different architectures such as matrix-type nanospheres (NS) [24], reservoir-type (core/shell) nanocapsules (NC) [25], as well as polymeric micelles [26]. In this study, in order to reach an optimized NP formulation for TQ, mPEG-PCL NPs were fabricated using different PCL chain lengths in the form of matrix-type NS or reservoir-type NC. The different formulations were evaluated for various physicochemical properties and in vitro release kinetics. Antiproliferative assays were then conducted in human cancer cell lines to investigate the anticancer activity of the optimized NP formulation. A pilot pharmacokinetic study was also carried out in a murine model to evaluate the oral bioavailability of the optimized TQ NP formulation.

## 2. Materials and Methods

### 2.1. Materials

Tin(II) 2-ethylhexanoate (stannous octoate), ε-caprolactone (CL), methoxy poly(ethylene glycol) (mPEG) molecular weight (MW) 5000, Span 80, and thymoquinone (TQ) were obtained from Sigma Aldrich (St. Louis, MO, USA). Castor oil was provided by Philadelphia Pharmaceuticals (Amman, Jordan). Polysorbate 80 (Tween 80,) was purchased from RFCL Ltd. (New Delhi, India). Absolute ethanol, acetone, methanol (HPLC grade), isopropanol, formic acid, water (HPLC grade), and dichloromethane (DCM) were obtained from Fisher Chemical (Thermo Fisher Scientific, Waltham, MA, USA). Diethyl ether and dimethyl sulfoxide were provided by Tedia (Fairfield, OH, USA). Phosphate buffered saline (PBS) 10×, pH 7.4, was obtained from Biowest (Nuaillé, France). Ultrapure water (~18.2 MΩ·cm) was prepared using a Millipore Direct-Q 5UV system (EMD Millipore, Billerica, MA, USA).

### 2.2. Synthesis of mPEG-PCL Copolymers

mPEG-PCL copolymers were synthesized by ring-opening polymerization of CL as previously described [24,26], where mPEG served as the macroinitiator and stannous octoate as the catalyst to form mPEG-PCL block copolymers. PCL chain length was varied by controlling the mPEG:CL feed ratio (Table S1). ^1^H-NMR in CDCl_3_ (Bruker 400 MHz instrument, Billerica, MA, USA) was used to confirm the copolymers’ structure and calculate the MW of the PCL block.

### 2.3. Preparation of TQ-Loaded NPs

Oil-filled core-shell TQ NC were prepared by nanoprecipitation as reported in our earlier publication [25]. The organic phase was a solvent mixture of acetone and ethanol (60:40, *v*/*v*) containing mPEG-PCL (25 mg), castor oil (150 µL), TQ (2.5 mg), and Span 80 (25 mg). The aqueous phase (10 mL) was composed of 0.2% Tween 80. The organic phase was added dropwise into the aqueous phase under moderate magnetic stirring and mixed overnight to evaporate the organic solvents and induce the formation of NC. Unencapsulated drug and excess excipients were removed by ultrafiltration (Pierce^TM^ Protein Concentrator, 100 kD molecular weight cut-off (MWCO), Thermo Scientific, Waltham, MA, USA) at 4000× *g* and 4 °C for 1 h (Hermle Z326K centrifuge, Wehingen, Germany), with repeated washing with ultrapure water twice. The final volume was completed to 10 mL with ultrapure water and the NP formulations were kept at 4 °C until further characterization. Matrix-type TQ NPs (NS) were prepared as described above without adding castor oil. The composition of the different TQ NPs prepared in this study is summarized in Table 1.

### 2.4. Characterization of TQ NPs by Dynamic Light Scattering (DLS) and Transmission Electron Microscopy (TEM)

For DLS measurements, freshly prepared formulations were diluted 1:1 with ultrapure water and analyzed using a Nicomp Nano Z3000 particle size/zeta potential instrument (Particle Sizing Systems, Santa Barbara, CA, USA). Measurements were reported from at least three different batches of each NP. For TEM imaging, one drop of NP dispersion was placed on Formvar-coated Cu grids (300 mesh, Electron Microscopy Sciences, Hatfield, PA, USA). After 1 min, excess liquid was blotted with the edge of a filter paper, followed by staining with uranyl acetate for 1 min and removing the excess liquid with filter paper. Imaging was performed on a Morgagni 268 TEM (FEI, Eindhoven, The Netherlands) at an accelerating voltage of 60 kV.

### 2.5. High-Performance Liquid Chromatography (HPLC) Analysis

The amount of TQ loaded in NPs and the amount released during in vitro release studies was analyzed by HPLC. The setup was composed of a Finnigan Surveyor LC Pump Plus system (Thermo Fisher Scientific, Waltham, MA, USA) equipped with an autosampler and a photodiode array UV detector. The mobile phase consisted of 0.1% formic acid, methanol, and isopropanol (50:45:5, *v*/*v/v*) at an isocratic flow rate of 1 mL min^−1^. Elution was performed on a C18 UniverSil column (5 μm, 150 × 4.6 mm; Fortis Technologies Ltd., Cheshire, UK) and the detection wavelength was set to 260 nm. TQ serial dilutions (0.16–20 μg mL^−1^) were prepared in the mobile phase and were used to construct the calibration curve, which was linear over the range of concentrations used (y = 218378x + 63037; R^2^ = 0.99733). The method was validated according to the International Conference on Harmonization (ICH) guidelines. Limit of quantitation (LOQ) and limit of detection (LOD) values were found to be 5.33 and 1.76 μg mL^−1^, respectively.

### 2.6. Determination of Drug Loading (DL) and Encapsulation Efficiency (EE)

For DL and EE determination, 0.1 mL of each freshly prepared NP dispersion was dissolved in 0.9 mL DMSO to ensure complete breakdown of the NPs. The samples were then diluted 1:1 in the HPLC mobile phase and analyzed as described above. The concentration of TQ was calculated based on a calibration curve of the peak areas of TQ standards versus concentration. Each measurement was performed in triplicate. DL and EE were calculated as follows:

DL (*w*/*w*) = Weight of loaded TQ/Weight of polymer (1)

EE (%) = (Weight of loaded TQ/Theoretical weight of TQ) × 100%(2)

### 2.7. Characterization by FT-IR

FT-IR spectra were obtained using an IR Affinity-1 spectrometer (Shimadzu, Kyoto, Japan). KBr discs were prepared with powdered samples of TQ and mPEG-PCL, or TQ NPs which were lyophilized using a FreeZone 4.5 L Benchtop freeze dryer (Labconco Corporation, Kansas City, MO, USA).

### 2.8. In Vitro Release of TQ from TQ-NC

TQ release was investigated in phosphate buffer (pH 7.4) and acetate buffer (pH 5.0) to simulate physiologic and lysosomal/tumor microenvironment pH, respectively. All release media contained 0.5% *w/v* Tween 80 to ensure sink conditions [25,26,27,28]. For the release test, triplicate samples of freshly prepared NPs (1 mL) were each transferred to a dialysis bag with 12–14 kD MWCO (Spectrum laboratories Inc., Rancho Dominguez, CA, USA) and immersed in 30 mL release media in tightly closed glass vials. Samples were placed in an orbital shaking incubator operating at 37 °C and 100 rpm (Biosan ES-20, Riga, Latvia). At predetermined time intervals, 10 mL of release media was collected and replaced with an equal volume of fresh media to maintain sink conditions. Samples collected were kept at 4 °C until analysis. TQ concentration was measured by HPLC under the conditions described above. Results were expressed as % cumulative amount of TQ released versus time.

### 2.9. Release Kinetics

The following kinetic models were applied in order to elucidate the mechanism of TQ release:

(3) $$\;\mathrm{Korsmeyer}–\mathrm{Peppas}:Q_{t}=k_{\mathrm{KP}}t^{n}$$

(4) $$\mathrm{Zero}\text{-}\mathrm{order}:\;Q_{t}=Q_{0}+k_{0}t$$

(5) $$\mathrm{First}\text{-}\mathrm{order}:\;Log\;Q_{t}=LogQ_{0}+k_{1}t/2.303$$

where *t* is the time, *Q_t_* is the % cumulative amount of drug released at time *t*, *k*_KP_ is the Korsmeye−Peppas rate constant, *n* is the release exponent, *Q*_0_ is the initial amount of drug released, *k*_0_ is the zero-order rate constant, and *k*_1_ is the first-order rate constant. Release data up to 60% cumulative release were fitted into each model.

### 2.10. In Vitro Antiproliferative Activity of TQ NPs

Antiproliferative activity was evaluated in various human cancer cell lines (MCF-7, PANC-1, and Caco-2) as well as a normal cell line (human dermal fibroblasts). All cell lines were obtained from the American Type Culture Collection (ATCC, Manassas, VA, USA). MCF-7, PANC-1, and Caco-2 cells were cultured in high glucose Dulbecco’s modified Eagle medium (DMEM; Eurobio Scientific, Les Ulis, France). Fibroblasts were cultured in Iscove’s modified Dulbecco’s medium (IMDM; Eurobio Scientific, Les Ulis, France). All media were supplemented with 10% fetal bovine serum, 1% L-glutamine, and 1% penicillin/streptomycin. The cell lines were maintained at 37 °C in a 5% CO_2_ atmosphere with 95% humidity. For the cytotoxicity experiments, cells were cultured in 96-well plates at density of 6 × 10^3^ cells per well to ensure exponential growth throughout the experimental period and to ensure a linear relationship between absorbance and cell number when analyzed by the sulforhodamine B (SRB) assay. After 24 h of seeding, cells were treated with free TQ (from a 20 mM stock solution in DMSO) and TQ NPs (F2-NC, equivalent to 1.38 mM TQ dispersed in PBS) at concentrations equivalent to 3.12, 6.25, 12.5, 25, 50, 100, and 200 µM TQ for 72 h. At the end of the incubation period, 200 µL of ice-cold 40% trichloroacetic acid (TCA) was added to each well, left at 4 °C for 1 h, and washed five times with distilled water. The TCA-fixed cells were stained for 30 min with 50 µL of 0.4% *w/v* SRB in 1% acetic acid. The plates were washed four times with 1% acetic acid and air dried for 30 min. One hundred microliters of 10 mM Tris base solution was then added to each well, and the absorbance was recorded on a microplate reader (BioTek Instruments, Winooski, VT, USA) at 570 nm. Results were expressed as % cell viability compared to the control (untreated cells). Each experiment was performed in triplicate.

### 2.11. Pilot Pharmacokinetic Study of TQ NPs after Oral Administration

Thirty male Balb/c mice (8–10 weeks old) were housed in plastic cages in an air-conditioned room with free access to food and water and were fasted overnight prior to the experiment. On the day of the experiment, animals were divided into two groups and each group was administered a single dose of 6 mg/kg TQ either as suspension in ultrapure water or in the form of TQ NPs (F2-NC) via oral gavage. At 1, 2, 4, 6, and 24 h post-administration, three animals from each group were randomly selected and euthanized by diethyl ether, and blood (~1 mL) was collected from the jugular vein into heparinized plastic tubes. Blood samples were left to sit for 45 min at room temperature then centrifuged at 5500 rpm for 30 min at room temperature. Approximately 0.5 mL of plasma was collected into microtubes, and plasma samples were stored at −80 °C until further analysis. The animal protocol for this work was approved by the Animal Care and Use Committee of Al-Zaytoonah University of Jordan (decision no. 1/5/2019–2020). All work was conducted in accordance with the Helsinki guidelines for animal research [29] and all applicable Jordanian governmental rules and guidelines.

### 2.12. Analysis of TQ Plasma Levels by Liquid Chromatography-Tandem Mass Spectrometry (LC-MS/MS)

#### 2.12.1. Instrumentation

The system consisted of a Shimadzu LCMS-8030 quadrupole mass spectrometer (Kyoto, Japan) equipped with an electrospray ionization (ESI) source, and a Shimadzu Prominence 30A Ultra-Fast Liquid Chromatography system composed of a system controller, degasser, binary pump and auto-sampler. Samples were eluted on a C8 column (5 μm, 50 × 2.1 mm) coupled with a guard column (5 μm, 10 × 2.1 mm) at 20 °C. The isocratic mobile phase consisted of acetonitrile (ACN) and 0.2% formic acid (50:50, *v*/*v*) at a flow rate of 0.2 mL min^−1^ and an injection volume of 20 μL. TQ samples were ionized by the ESI source in negative ion mode (5.5 eV spray voltage). The dwell time was set to 50 ms and the probe temperature was 400 °C. Quantification was performed using multiple reaction monitoring (MRM) of the transitions at *m/z* 164.0→134.1.

#### 2.12.2. Preparation of Calibration Curve Samples

TQ was dissolved in ACN to make a stock solution at 1 mg mL^−1^. TQ working standard solutions were prepared by serial dilutions of the stock solution in the mobile phase. Calibration curve samples were prepared by spiking the appropriate TQ working standard solutions into blank plasma obtained from untreated mice to achieve concentrations from 2 to 1000 ng mL^−1^.

#### 2.12.3. Preparation of Plasma Samples

To 100 μL of plasma (spiked with calibration curve samples or plasma extracted from treated mice), ethyl acetate (1 mL) was added and vortex mixed for 30 s. The solutions were then centrifuged at 14,000 rpm, and the supernatant was separated, dried, and reconstituted in 100 μL of the mobile phase. Samples were then injected into the LC-MS/MS system to quantify the plasma concentration of TQ at each time point. Pharmacokinetic parameters including the maximum plasma concentration (C_max_), time to achieve C_max_ (*t*_max_), plasma half-life (*t*_1/2_), and the area under the curve (AUC_0→__∞_) were deduced from the plasma concentration versus time plots using Graphpad Prism 6.0e. Plasma *t*_1/2_ was determined by fitting the elimination phase of the plots to the first-order model.

### 2.13. Statistical Analysis

Results were analyzed using Graphpad Prism 6.0e. All values were reported as mean ± SD. Statistical differences were examined by one-way or two-way analysis of variance (ANOVA) as appropriate, followed by Tukey or Sidak’s multiple comparisons tests, respectively, where *p* < 0.05 was considered statistically significant.

## 3. Results and Discussion

### 3.1. Preparation and Characterization of TQ-Loaded NPs with Varying Polymer MW and NP Architectures

mPEG-PCL copolymers with two different PCL chain lengths were synthesized in this study by controlling the mPEG:CL weight ratio during synthesis. The structure of the copolymers and MW of PCL was confirmed using ^1^H-NMR (Figure S1) by relying on the relative integration ratios between the proton peaks referring to the ethylene oxide repeating units and any one of the proton peaks of CL repeats [26]. Accordingly, when the feed ratio of mPEG:CL was 1:2, the MW of PCL was calculated to be 10,364 Da. A feed ratio of 1:4 produced a copolymer with a PCL MW of 18,506 Da. The two copolymers were referred to as mPEG5K-PCL10.3K and mPEG5K-PCL18.5K, respectively (Table S1).

**Figure 1:**
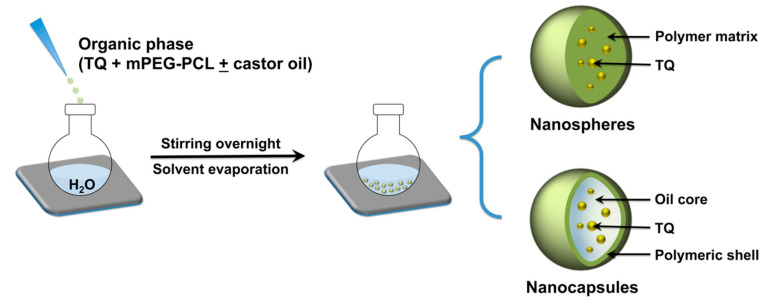
Overview of TQ NP fabrication by nanoprecipitation using mPEG-PCL. Different formulations were prepared by varying the MW of the PCL block with or without the addition of castor oil to the organic phase, which resulted in the formation of matrix-like nanospheres (NS) or reservoir-type nanocapsules (NC) with an oil-filled core and a polymeric shell.

mPEG-PCL copolymers were employed to fabricate TQ-loaded NPs by the nanoprecipitation technique. An overview of the NP preparation process is presented in Figure 1. This technique is typically used to fabricate matrix-type NPs where the loaded drug becomes entrapped within the polymer matrix as the organic phase is slowly added to the aqueous phase. The method can be adapted to produce reservoir-type NPs by incorporating oils in the organic phase [30]. With the appropriate combination of lipophilic and hydrophilic surfactants in the organic and aqueous phases, respectively, the procedure can lead to the formation of drug-loaded NPs with a core/shell architecture where the drug becomes entrapped in the oil core [31,32]. Various vegetable oils and triglycerides may be employed to form oil-cored NC [32]. In this study, castor oil was used as it has previously demonstrated superior ability to solubilize various polyphenolic and aromatic drug molecules during NC formation [25,33,34].

**Figure 2:**
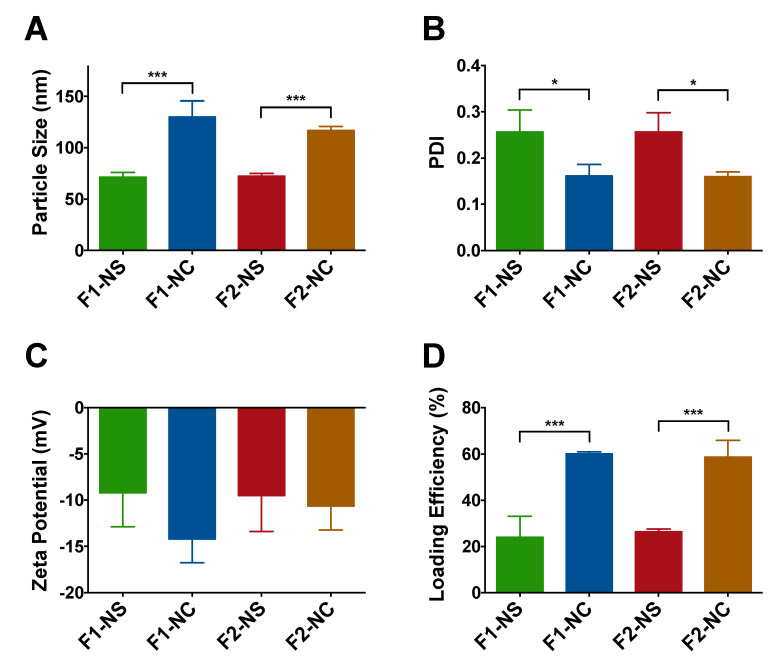
Characterization of TQ NPs prepared in this study. (**A**) Particle size of NC-based formulations F1-NC and F2-NC was 1.8 and 1.6 fold larger, respectively, compared to the NS-based formulations F1-NS and F2-NS due to the presence of the oil core; (**B**) NC-based formulations were associated with significantly lower polydispersity index (PDI) values, indicating greater monodispersity compared to their NS-based counterparts; (**C**) all NP formulations displayed partially negative zeta potential values; (**D**) the NC architecture is more favorable for TQ loading as evidenced by the significant increase in loading efficiency by 2.5 and 2.2 fold in F1-NC and F2-NC, respectively, compared to F1-NS and F2-NS. All results are presented as mean ± SD from at least three different batches of NPs. * *p* < 0.05, *** *p* < 0.001.

The different NP formulations were characterized in terms of particle size, polydispersity, zeta potential, and drug loading efficiency (Figure 2 and Table S2). As shown in Figure 2A, particle size of the formulations was not impacted by the PCL MW, rather it showed strong dependence on the NP architecture. While both NS formulations F1-NS and F2-NS exhibited a similar particle size of 72 nm, the NC formulations F1-NC and F2-NC were significantly larger (*p* < 0.001) with an average particle size of 130 and 117 nm, respectively. Since the only difference between NS and NC formulations was the addition of castor oil, the increase in particle size may be attributed to the oil core−polymer shell architecture of NC. PDI values were used as an indicator of NP homogeneity in terms of size. As can be seen in Figure 2B, NC formulations were associated with PDI values of 0.17 and 0.16 for F1-NC and F2-NC, respectively. The NS formulations were significantly more polydisperse (*p* < 0.05) with average PDI values of 0.26 for both F1-NS and F2-NS. These findings are consistent with a previous report from our group using a similar combination of polymers, surfactants, and oil [25], and further confirm the ability of the NC architecture to produce highly monodisperse NPs. The zeta potential values for all formulations were partially negative and ranged between −9.2 to −14.2 mV (Figure 2C). Although the magnitude of the surface charge may not seem sufficient to impart colloidal stability, the presence of the PEG corona on the NP surface can effectively maintain steric stability and prevent NP aggregation, as evidenced by results from a previously reported stability study using a similar NP platform [25]. Moreover, the MW for PEG used in this study was 5000, which is within the MW range typically employed to achieve effective anti-fouling for nanocarriers [35].

**Figure 3:**
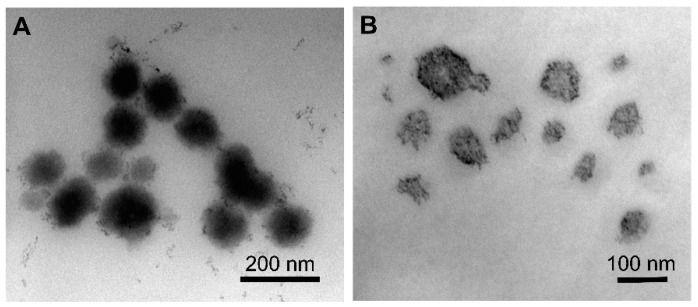
TEM images of (**A**) F2-NC and (**B**) F2-NS. F2-NC is characterized by a spherical morphology and core/shell architecture appearing as a dark core surrounded by a lighter outer layer, whereas F2-NS appeared as polydisperse matrix-like particles.

The drug loading capacity of TQ NPs was found to be highly dependent on the NP architecture, while no difference in loading was observed between NPs with the same PCL MW (Figure 2D). NC formulations F1-NC and F2-NC were able to achieve 60.1 and 58.7% loading efficiencies, respectively, which were 2.5 and 2.2 times higher than the loading efficiencies obtained with F1-NS and F2-NS (24.0 and 26.3%, respectively). Again, the difference may be attributed to the presence of the oil-filled core in NC formulations, which has been shown to improve the NP loading capacity for lipophilic compounds compared to matrix-like NPs [33,36]. Based on these findings, it was concluded that the NC architecture was a more promising NP platform for TQ, and the NS formulations were excluded from subsequent investigations. TEM imaging of F2-NC (Figure 3A) showed a spherical morphology unlike F2-NS (Figure 3B), which appeared as polydisperse particles that were less uniform in shape. In addition, negative staining of the NC allowed the observation of the core/shell architecture, with the polymeric shell appearing as a light grey outer layer surrounding a darker core. On the other hand, F2-NS particles were characterized by a diffuse gray color with no discernible regions indicating their matrix-like architecture.

**Figure 4:**
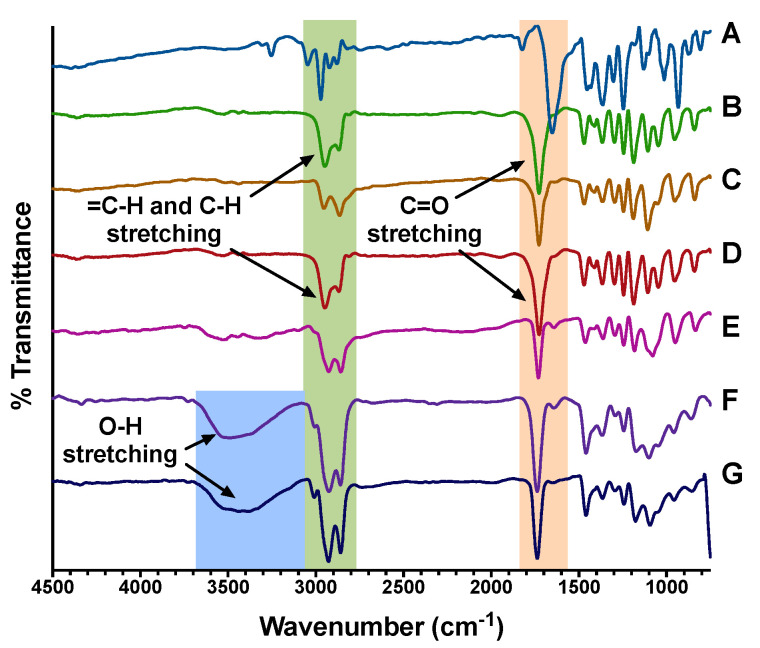
FT-IR spectra of (**A**) TQ, (**B**) mPEG5K-PCL10.3K, (**C**) mPEG5K-PCL18.5K, (**D**) F1-NS, (**E**) F2-NS, (**F**) F1-NC, and (**G**) F2-NC. TQ exhibited =C–H, C–H, and C=O stretching at 3050 cm^−1^, 2970 cm^−1^, and 1655 cm^−1^, respectively. mPEG-PCL copolymers showed C–H stretching vibrations at 2950 and 2864 cm^−1^ attributed to the ethylene oxide repeats, and a sharp C=O stretching band at 1730 cm^−1^ corresponding to the carbonyl ester groups of PCL. The broad O–H stretching band appearing between 3700–3100 in the NC formulations (**F**) and (**G**) indicates the presence of hydrogen bonding interactions between the formulation components after adding castor oil.

The NC formulations were further characterized by FT-IR spectroscopy. As shown in Figure 4, the FT-IR spectrum of TQ revealed characteristic bands corresponding to =C–H, C–H, and C=O stretching at 3050, 2970, and 1655 cm^−1^, respectively. mPEG-PCL copolymers exhibited C–H stretching vibrations at 2950 and 2864 cm^−1^ which were attributed to the ethylene oxide repeats, and a sharp C=O stretching band at 1730 cm^−1^ corresponding to the carbonyl ester groups of the PCL repeats. Upon encapsulation of TQ to form F1-NC and F2-NC, several spectral changes were observed. A broad O–H stretching band appeared between 3700–3100 cm^−1^ in the NC formulations but was absent from the NS formulations F1-NS and F2-NS. This indicates the presence of hydrogen bonding interactions between the formulation components after adding castor oil which has three –OH groups. A similar observation was obtained when encapsulating the polyphenolic compound cirsiliol in castor oil-filled mPEG-PCL NC [25]. In addition, the =C–H stretching band of TQ was shifted from 3050 to 3014 cm^−1^ after encapsulation. This shift was accompanied by an increase in the intensity of C–H stretching vibrations between 3030–2780 cm^−1^. The C=O bands of TQ and mPEG-PCL copolymers which originally appeared at 1655 and 1730 cm^−1^, respectively, were also shifted to a single sharp band appearing at 1740 cm^−1^. The spectral changes strongly indicate the compatibility of the formulation components enabled by intermolecular interactions such as H-bonding in addition to van der Waal forces.

### 3.2. Effect of PCL MW and pH of the Release Medium on TQ Release

**Figure 5:**
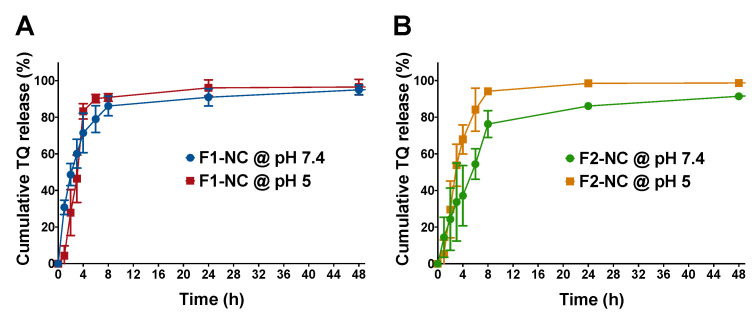
In Vitro release of TQ NPs conducted in phosphate buffered saline (PBS) (pH 7.4) and acetate buffer (pH 5.0). Results are presented as mean ± SD of cumulative % amount of TQ released from (**A**) F1-NC and (**B**) F2-NC up to 48 h. The PCL MW impacted the release kinetics. F1-NC with a shorter PCL chain achieved 86–90% cumulative release within the first 8 h in neutral and acidic media, respectively. Conversely, TQ release from F2-NC with the longer PCL chain was more sustained at pH 7.4, reaching 76% cumulative release within the first 8 h and 86% release after 24 h, which was accelerated in acidic media to 94 and 99% at 8 and 24 h, respectively.

In Vitro release of TQ from NC formulations F1-NC and F2-NC was investigated in PBS at pH 7.4 and in acetate buffer at pH 5.0 to mimic physiologic and lysosomal/tumor microenvironment pH, respectively. Samples were placed in dialysis membranes with MWCO 12–14 kD, which have previously been shown to allow free diffusion of small drug molecules with minimal effect on controlling drug release [25]. Samples were periodically withdrawn from the release media and were analyzed for TQ content by HPLC. The values were then used to construct the release profiles by plotting the cumulative % amount of TQ released from each formulation versus time. Release profiles of TQ from F1-NC at pH 7.4 and pH 5.0 are depicted in Figure 5A, whereas the release profiles corresponding to F2-NC at the same pH conditions are shown in Figure 5B. Both formulations resulted in the typical biphasic release profiles associated with polymeric delivery systems, with relatively fast release within the first 8 h and more sustained release up to 48 h. However, release was much faster in case of F1-NC, which achieved 86–90% cumulative release within the first 8 h in neutral and acidic media, respectively. In addition, the release profiles at the different pH conditions were superimposable. Conversely, TQ release from F2-NC was more sustained at pH 7.4, attaining 76% cumulative release within the first 8 h and 86% release after 24 h, which was accelerated in acidic media to reach 94 and 99% after 8 and 24 h, respectively. The difference in release kinetics was most likely attributed to the difference in MW of the biodegradable PCL. In the case of F1-NC, which was prepared using mPEG5K-PCL10.3K, the degradation of the PCL chains was relatively fast, even at neutral pH, resulting in a release profile similar to that obtained at pH 5.0. On the other hand, F2-NC prepared using mPEG5K-PCL18.5K was able to achieve better control over TQ release at pH 7.4 and faster release at pH 5, which is more favorable for in vivo applications.

TQ release kinetics as a function of PCL MW and pH conditions were further examined by fitting the release data up to 60% cumulative release to various kinetic models typically applied in polymeric systems, such as the Korsmeyer–Peppas, zero-order, and first-order models. Kinetic parameters were calculated and were used to find the best-fit model for each formulation/condition in order to better understand the mechanism of drug release. As summarized in Table S3, the goodness-of-fit for each formulation and pH condition was found based on the value of the coefficient of determination (*R*^2^). Both F1-NC and F2-NC were best fitted to the Korsmeyer–Peppas model when release was conducted at pH 7.4 and the zero-order model when release was conducted at pH 5.0. At pH 7.4, F1-NC was associated with a release rate constant *k*_KP_ value of 31.0, which was greater than *k*_KP_ for F2-NC (14.6). The difference in *k*_KP_ between the two formulations correlates well with the faster release kinetics observed in F1-NC compared to F2-NC. The value of the release exponent *n* was also used to elucidate the mechanism of TQ release from the NP formulations. When the Korsmeyer–Peppas model is applied to spherical particles, a value of *n* = 0.43 indicates drug release by Fickian diffusion. On the other hand, a value of *n* between 0.43 and 0.85 is indicative of anomalous or non-Fickian transport. In this case, drug release occurs through a combination of simple diffusion and polymer chain relaxation. A value of *n* ≥ 0.85 indicates supercase II transport, where drug release is primarily governed by polymer relaxation [37,38]. In this study, the value of *n* for TQ NPs at pH 7.4 was found to be 0.6 and 0.7 for F1-NC and F2-NC, respectively, signifying a similar release mechanism: anomalous transport. This mechanism is expected from core/shell systems when the shell is comprised of a biodegradable polymer. In the context of this study, TQ release most likely began by diffusion away from the oil core and across the polymeric shell into the release medium, while the polymeric shell was undergoing degradation and chain relaxation, which was faster in the case of F1-NC due to the shorter PCL chain. Interestingly, a recent study from our group reported much slower release kinetics for the flavonoid cirsiliol from a formulation similar to F2-NC, with ~20% cumulative release achieved within the first 48 h, and ~40% release attained after 96 h [25]. The difference may be attributed to the relatively small size of TQ (MW 164.2 g/mol) compared to cirsiliol (MW 330.1 g/mol), which allowed it to diffuse out of the NC formulation more freely.

When release was carried out at pH 5.0, both formulations exhibited zero-order kinetics, indicating constant TQ release from the NC core. F2-NC was associated with a release rate constant *k*_0_ of 24.2 h^−1^, whereas *k*_0_ for F1-NC was slightly lower (21.0 h^−1^). However, the two release profiles were similar across all time points (Figure 5A,B). The observed similarity in release kinetics at pH 5.0 indicates that the low pH of the release medium caused an acceleration in the degradation rate of mPEG5K-PCL18.5K, resulting in a similar contribution to controlling TQ release as that of mPEG5K-PCL10.3K. Based on these results, F2-NC was chosen for subsequent biological assays, having shown better control over release kinetics and pH-sensitive drug release.

### 3.3. Antiproliferative Activity of TQ-Loaded NPs in Human Cancer and Normal Cell Lines

**Figure 6:**
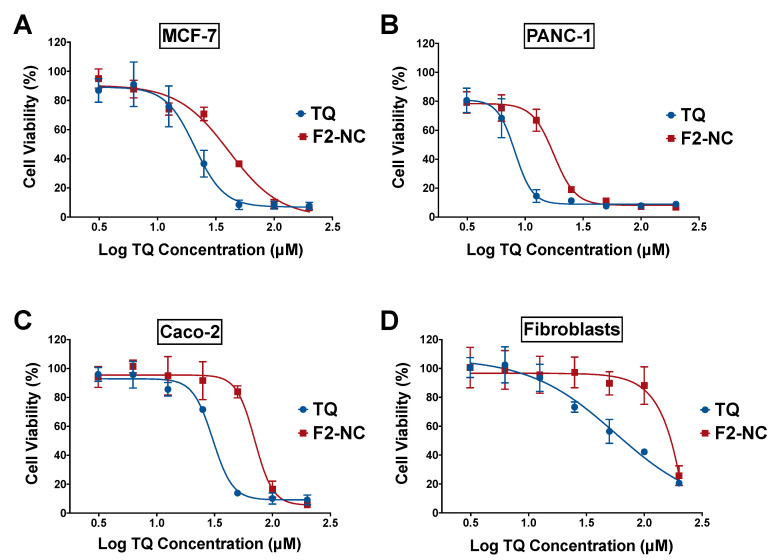
Antiproliferative activity of free TQ and the optimized TQ NP formulation F2-NC in (**A**) MCF-7, (**B**) PANC-1, and (**C**) Caco-2 human cancer cell lines, as well as (**D**) human dermal fibroblasts serving as a model normal cell line. Cells were incubated with various concentrations of TQ in its free or NP form in complete media for 72 h. Cell viability was measured via a sulforhodamine B (SRB) assay and expressed relative to untreated controls. TQ and F2-NC exhibited dose-dependent cytotoxicity with micromolar potencies across all cancer cell lines. Although free TQ was more potent than F2-NC, the latter was significantly less toxic to fibroblasts and was associated with a greater selectivity index.

Several mechanisms have been proposed for TQ’s anticancer effects in various cell lines and animal models [39]. TQ has been shown to exert its cytotoxic effects by modulating multiple cancer hallmarks such as proliferation, cell cycle progression, invasion and metastasis, tumor-induced inflammation, and induction of angiogenesis [7]. In this study, the optimized TQ NP formulation (F2-NC) was examined for its antiproliferative activity in order to verify its potential to serve as an anticancer nanomedicine. In vitro assays were conducted in cell culture monolayers to screen the anticancer activity of the developed formulation and form the basis for future studies involving mechanistic understanding of the anticancer effects in vitro and in vivo. Assays were performed on MCF-7, PANC-1, and Caco-2 human cancer cell lines, with human dermal fibroblasts serving as a normal cell line to determine the NP’s anticancer selectivity. The blank drug-free NC formulation was previously tested against cancer and normal cell lines and was shown to be nontoxic [25]. Cells were incubated with various concentrations of free TQ and F2-NC for 72 h, followed by assessing cell viability via an SRB assay. As depicted in Figure 6, free TQ and TQ NPs exhibited a dose-dependent inhibitory effect on cancer cell growth and the concentrations needed to achieve 50% growth inhibition (IC_50_) were in the micromolar range (Table 2). The following order of potency was obtained for both TQ and F2-NC: PANC-1 > MCF-7 > Caco-2.

In general, free TQ was approximately two-fold more potent than F2-NC, which may be attributed to differences in cellular uptake and intracellular release kinetics of TQ from F2-NC. Note that previously reported NP formulations of TQ did not always result in potentiation of its anticancer activity. For example, TQ-loaded PEGylated polymeric NPs were more effective in killing breast cancer cells compared to the free drug [19]. On the other hand, Fakhoury et al. reported that TQ NPs exhibited equal or more potent anticancer activity compared to free TQ depending on the cancer model [18]. Moreover, a recent study by Ramzy et al. described TQ-encapsulated polymeric NPs targeted to colon cancer cells where the NPs were significantly less potent than free TQ due to sustained drug release [40].

Anticancer selectivity for free TQ and F2-NC was determined by the selectivity index (SI), which was calculated by dividing the IC_50_ value obtained in fibroblasts for TQ and F2-NC by the IC_50_ obtained in each cancer cell line. As shown in Table 2, the NP formulation exhibited superior anticancer selectivity compared to free TQ, particularly in PANC-1 cells where the SI for F2-NC was found to be 64.0 compared to 6.7 for free TQ. These results indicate that despite the lower potency for F2-NC, it may be more effective therapeutically than free TQ due to the enhanced cancer cell selectivity. These results were consistent with a recent report by Shahein et al., where TQ-loaded mesoporous silica NPs improved the drug’s targeting efficacy toward cancer cells by exhibiting low toxicity to normal cells unlike free TQ [41]. Having shown the highest potency and the greatest selectivity against PANC-1 cells, our in vitro findings strongly support moving forward with F2-NC as a nanomedicine against pancreatic tumors in a suitable in vivo model.

### 3.4. TQ-Loaded NPs Are a Promising Strategy to Increase TQ’s Oral Bioavailability

**Figure 7:**
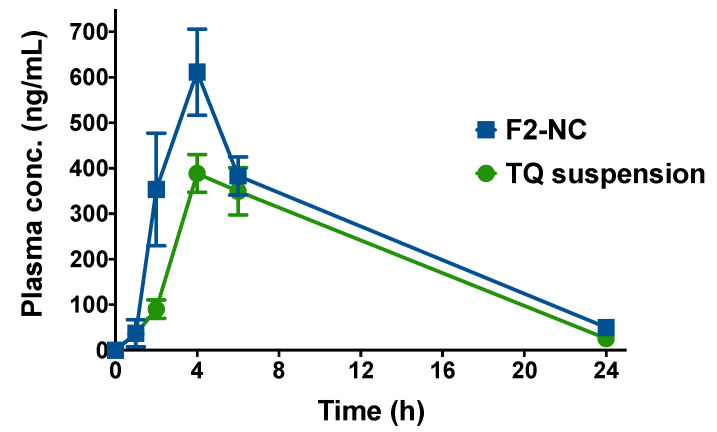
Plasma concentration versus time profiles following oral administration of TQ suspension or TQ NPs (F2-NC) to male Balb/c mice. Animals were divided into two groups (TQ suspension or F2-NC) and each group was given a dose equivalent to 6 mg/kg TQ via oral gavage. At 1, 2, 4, 6, and 24 h post-administration, three animals from each group were sacrificed and the plasma concentration of TQ was analyzed by LC-MS/MS to construct the plasma profile. Although both groups exhibited the same *t*_max_ of 4 h, F2-NC was able to achieve a significantly higher C_max_ compared to TQ suspension and a 1.3-fold increase in AUC, indicating superior bioavailability of the TQ NP formulation.

A pilot in vivo study was conducted in mice in order to evaluate the ability of the optimized TQ NP formulation to enhance the rate and extent of TQ absorption after oral administration. Animals were administered an oral dose equivalent to 6 mg/kg TQ either as aqueous suspension or in the form of F2-NC. At different time points, animals were euthanized and the plasma concentration of TQ was determined by LC-MS/MS. The resultant plasma concentration versus time profiles are shown in Figure 7, and the calculated pharmacokinetic parameters are summarized in Table 3.

TQ suspension and F2-NC were both associated with a similar *t*_max_ of 4 h, indicating equal rates of absorption. However, F2-NC was able to achieve a significantly higher plasma concentration at 2 h (*p* < 0.0001). The NP formulation was also associated with a significantly greater C_max_ of 611.4 ng mL^−1^ compared to only 388.5 ng mL^−1^ for TQ suspension (*p* < 0.001). Consequently, F2-NC (AUC: 6069 ng h mL^−1^) resulted in a 1.3-fold enhancement in bioavailability (*p* < 0.05) compared to TQ suspension (AUC: 4669 ng h mL^−1^). Even though the elimination *t*_1/2_ was slightly longer for F2-NC compared to TQ suspension (5.8 and 5.0 h, respectively), they were not significantly different. Taken together, these findings strongly indicate that F2-NC is readily absorbed from the gastrointestinal tract and can reach the systemic circulation to a greater extent compared to TQ suspension. This is largely attributed to the increased solubility of TQ when formulated as F2-NC compared to the poorly soluble free drug, consistent with previous reports involving TQ NP formulations [12,13,14]. Enhancing the solubility of hydrophobic drug molecules has been a leading motivation for pharmaceutical applications of nanotechnology. Based on the modest difference in *t*_1/2_, it is likely that TQ is liberated from F2-NC during oral absorption and reaches the systemic circulation in its free form, resulting in similar elimination kinetics as TQ suspension.

## 4. Conclusions

In this study, we showed that NPs based on the biodegradable copolymer mPEG-PCL could be effectively tailored to design a nanoscale delivery system for the bioactive compound TQ. The optimal TQ NP formulation was found to be in the form of castor oil-filled NC based on the mPEG-PCL copolymer with the relatively larger PCL MW (F2-NC). F2-NC was associated with a monodisperse particle size, very good loading efficiency, spherical morphology, and sustained release properties. Even though the NC formulation exhibited lower anticancer potency compared to the free drug, it was associated with significantly greater selectivity, supporting its utility as an anticancer nanomedicine, particularly against pancreatic cancer where it showed the best selectivity. Preliminary pharmacokinetic evaluation in mice revealed an enhancement in oral bioavailability for F2-NC compared to free TQ, signifying its ability to enhance the biopharmaceutical properties of TQ.

## Figures and Tables

**Table 1:** Composition of thymoquinone nanoparticles (TQ NPs) prepared in this study.

Formulation	Organic Phase ^a^			Aqueous Phase ^b^
	Copolymer Code	Castor Oil (µL)	Span 80 (mg)	Tween 80 (% *w*/*v*)
F1-NS ^c^	mPEG5K-PCL10.3K	-	25	0.2
F1-NC ^d^	mPEG5K-PCL10.3K	150	25	0.2
F2-NS	mPEG5K-PCL18.5K	-	25	0.2
F2-NC	mPEG5K-PCL18.5K	150	25	0.2

^a^ Composed of 5 mL acetone/ethanol (60:40, *v*/*v*); ^b^ Composed of 10 mL ultrapure water; ^c^ NS: nanospheres; ^d^ NC: nanocapsules.

**Table 2:** Summary of IC_50_ values and selectivity indices (SI) for TQ and F2-NC.

Cell Line	TQ		F2-NC	
	IC_50_ (µM)	SI ^a^	IC_50_ (µM)	SI
MCF-7	20.5 ± 3.7	2.9	40.6 ± 2.1	27.9
PANC-1	8.8 ± 2.2	6.7	17.6 ± 1.2	64.0
Caco-2	30.4 ± 0.5	1.9	70.4 ± 0.2	16.0
Fibroblasts	58.9 ± 10.3	-	1129.5 ± 47.4	-

^a^ Calculated by dividing the IC_50_ value obtained in human dermal fibroblasts by the IC_50_ value obtained in each cancer cell line.

**Table 3:** Pharmacokinetic parameters following oral administration of TQ suspension and F2-NC. Results are presented as mean ± SD (*n* = 3).

Parameter	TQ Suspension	F2-NC
C_max_ (ng mL^−1^)	388.5 ± 41.5	611.4 ± 94.9
*t*_max_ (h)	4.0	4.0
*t*_1/2_ (h)	5.0	5.8
AUC_0→__∞_ (ng h mL^−1^)	4669 ± 508	6069 ± 492

## Supplementary Materials

The following are available online at https://www.mdpi.com/1999-4923/12/9/811/s1, Figure S1: ^1^H-NMR spectra of mPEG-PCL copolymers, Table S1: Characterization of the copolymers synthesized in this study, Table S2: Characterization of TQ NPs prepared in this study, and Table S3: Kinetic parameters for TQ release from F1-NC and F2-NC.
