# Supplementary material for: Development of a Thymoquinone Polymeric Anticancer Nanomedicine through Optimization of Polymer Molecular Weight and Nanoparticle Architecture
Source: Pharmaceutics. 2020 Aug 27;12(9):811. doi: 10.3390/pharmaceutics12090811 (PMC7560238; doi:10.3390/pharmaceutics12090811)
Supplement: Supplementary file 1 [file pharmaceutics-12-00811-s001.pdf]

pharmaceutics

Supplementary Materials: Development of a Thymoquinone Polymeric Anticancer Nanomedicine through Optimization of Polymer Molecular Weight and Nanoparticle Architecture

Suhair Sunoqrot, Malek Farraj, Ala'a M. Hammad, Violet Kasabri, Dana Shalabi, Ahmad A. Deeb, Lina Hasan Ibrahim, Khaldoun Shnewer and Ismail Yousef

Table S1. Characterization of the copolymers synthesized in this study.

Copolymer	mPEG: CL	MW of PCL by ^{1}H-NMR (g/mol)
mPEG5K-PCL10.3K	1:2	10,364
mPEG5K-PCL18.5K	1:4	18,506

Table S2. Characterization of TQ NPs prepared in this study (values correspond to Figure 2 of the main text).

Formulation	Particle Size (nm)	PDI	Zeta Potential (mV)	Loading (µg TQ/Mg Polymer)	Loading Efficiency (%)
F1-NS	72 ± 4	0.26 ± 0.05	−9.2 ± 3.7	24.0 ± 9.0	24.0 ± 9.0
F1-NC	130 ± 16	0.17 ± 0.03	−14.2 ± 2.6	60.1 ± 0.9	60.1 ± 0.9
F2-NS	72 ± 3	0.26 ± 0.04	−9.5 ± 3.9	26.3 ± 1.2	26.3 ± 1.2
F2-NC	117 ± 4	0.16 ± 0.01	−10.6 ± 2.6	58.7 ± 7.2	58.7 ± 7.2

Table S3. Kinetic parameters for TQ release from F1-NC and F2-NC obtained by fitting in vitro release data to different kinetic models of drug release (Equations (3)–(5)). The best-fit models for each formulation are highlighted in yellow.

Formulation	Release Medium	Korsmeyer–Peppas			Zero-Order		First-Order	
		R^2	k_{KP}	n	R^2	k_0	R^2	k_1
F1-NC	pH 7.4	0.99655	31.076	0.6135	0.98443	14.641	0.95703	0.334
	pH 5.0	0.97243	4.8104	2.1978	0.99528	20.993	0.90291	1.1766
F2-NC	pH 7.4	0.99288	14.604	0.7223	0.98679	7.7204	0.92006	0.2478
	pH 5.0	0.98520	5.7026	2.1383	1.0000	24.218	0.92782	1.1528

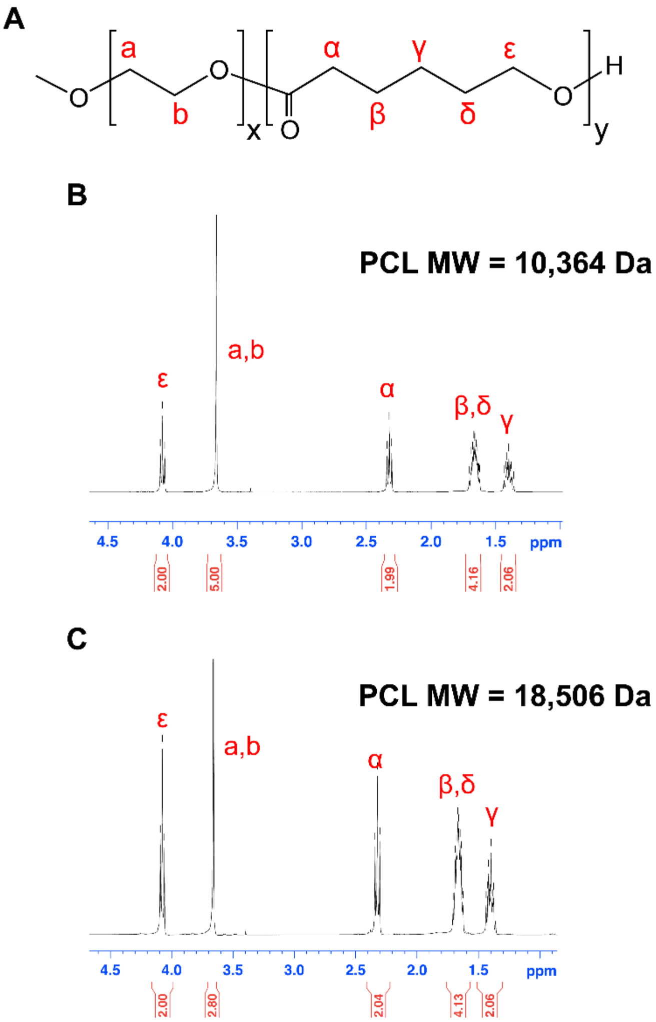

Figure S1. Confirmation of mPEG-PCL structure and MW by ^{1}H-NMR. (**A**) Chemical structure of mPEG-PCL; (**B**) ^{1}H-NMR spectrum of mPEG5K-PCL10.3K synthesized at an mPEG:CL feed ratio of 1:2; (**C**) ^{1}H-NMR spectrum of mPEG5K-PCL18.5K synthesized at an mPEG:CL feed ratio of 1:4. The MW of the PCL blocks was calculated based on the relative integration ratio of the ethylene oxide protons (a,b) to any one of the PCL protons (α, β, γ, δ, or ε).
